# Biallelic loss-of-function variants in *WDR11* are associated with microcephaly and intellectual disability

**DOI:** 10.1038/s41431-021-00943-5

**Published:** 2021-08-20

**Authors:** Natja Haag, Ene-Choo Tan, Matthias Begemann, Lars Buschmann, Florian Kraft, Petra Holschbach, Angeline H. M. Lai, Maggie Brett, Ganeshwaran H. Mochida, Stephanie DiTroia, Lynn Pais, Jennifer E. Neil, Muna Al-Saffar, Laila Bastaki, Christopher A. Walsh, Ingo Kurth, Cordula Knopp

**Affiliations:** 1 Institute of Human Genetics, Medical Faculty, RWTH Aachen University, Aachen, Germany; 2 KK Research Centre/Genetics Service, KK Women’s & Children’s Hospital, Singapore, Singapore; 3 Division of Neuropediatrics and Social Pediatrics, Department of Pediatrics, Medical Faculty, RWTH Aachen University, Aachen, Germany; 4 Division of Genetics and Genomics, Boston Children’s Hospital, and Departments of Pediatrics and Neurology, Harvard Medical School, Boston, MA USA; 5 Center for Mendelian Genomics, Program in Medical and Population Genetics, Broad Institute of MIT and Harvard, Cambridge, MA USA; 6 Howard Hughes Medical Institute, Boston Children’s Hospital, Boston, MA USA; 7 Department of Paediatrics, College of Medical and Health Sciences, United Arab Emirates University, Al Ain, UAE; 8 Kuwait Medical Genetics Centre, Maternity Hospital, Shuwaikh, Kuwait

**Keywords:** Disease genetics, Diseases

## Abstract

Heterozygous missense variants in the *WD repeat domain 11* (*WDR11*) gene are associated with hypogonadotropic hypogonadism in humans. In contrast, knockout of both alleles of *Wdr11* in mice results in a more severe phenotype with growth and developmental delay, features of holoprosencephaly, heart defects and reproductive disorders. Similar developmental defects known to be associated with aberrant hedgehog signaling and ciliogenesis have been found in zebrafish after *Wdr11* knockdown. We here report biallelic loss-of-function variants in the *WDR11* gene in six patients from three independent families with intellectual disability, microcephaly and short stature. The findings suggest that biallelic *WDR11* variants in humans result in an overlapping but milder phenotype compared to *Wdr11*-deficient animals. However, the observed human phenotype differs significantly from dominantly inherited variants leading to hypogonadotropic hypogonadism, suggesting that recessive *WDR11* variants result in a clinically distinct entity.

## Introduction

*WDR11* encodes for the WD repeat domain 11  protein and has been shown to be broadly expressed in the developing central nervous system of mice [1]. In humans, heterozygous missense variants in the *WDR11* gene have been associated with hypogonadotropic hypogonadism type 14 with or without anosmia (OMIM #614858) [1]. Congenital hypogonadotropic hypogonadism (CHH) is clinically characterized by pubertal failure and infertility due to deficient production, secretion or action of gonadotropin-releasing hormone (GnRH) and can be associated with various additional developmental defects [2]. When associated with reduced or lack of ability to smell (hyposmia or anosmia), CHH is termed Kallmann syndrome. Animal studies showed that Hedgehog (Hh) signaling in the primary cilium was impaired by loss of WDR11 [3]. *Wdr11*-defective mice and zebrafish demonstrated complex developmental abnormalities indicative for aberrant Hh signaling and impaired ciliogenesis [3]. The associated phenotype in *Wdr11*-null mice comprised features of holoprosencephaly such as microcephaly, hypotelorism, microphthalmia/anophthalmia, dysmorphogenesis of the pituitary gland and growth retardation, as well as heart defects, infertility and hypoplasia of reproductive organs. Mouse Embryonic Fibroblasts (MEFs) from *Wdr11*-null mice showed a reduced length of the ciliary axoneme and reduced frequency of ciliated cells, further supporting the essential role of *WDR11* for ciliogenesis. A similar effect was seen in a human embryonic olfactory GnRH neuroblast cell line after knockdown of endogenous *WDR11* [3]. However, no human phenotype associated with biallelic variants in *WDR11* has been described yet. In this study, we report biallelic loss-of-function variants in six affected individuals from three families who presented with intellectual disability, microcephaly and mild short stature.

## Material and methods

### Genetic investigation

Written informed consent was obtained from all participating family members of the three families. Whole-exome sequencing (WES) in family A, individual III-1 and III-2 was performed at the Institute of Human Genetics, RWTH Aachen University, Medical Faculty, as previously described [4]. GeneMatcher [5] facilitated identification of two other families (B and C) with homozygous loss-of-function variants in *WDR11*. Clinical exome sequencing (Illumina TruSight One sequencing panel) was performed in individual II-I of family B. For family C, WES was conducted on a research basis on individuals III-1, III-4 and III-5. Sanger sequencing was performed in parents and all affected and unaffected siblings of all included families. The identified variants in *WDR11* were deposited in ClinVar (https://www.ncbi.nlm.nih.gov/clinvar/) with accession numbers: SCV001593158, SCV001593159, SCV001593160, and SCV001593161.

### Western blot

Protein isolation from cultured fibroblasts of individual III-1 from family A and Western blot were carried out as described [6]. The primary antibodies used for immunodetection were rabbit anti-WDR11 (Novus Biologicals, NBP1-89930, 1:500, N-terminal epitope aa 268-348) and rabbit anti-WDR11 (Abcam, ab93871, 1:500, C-terminal epitope aa 1174–1224). Rabbit anti-α-Tubulin (Abcam, ab15246) and mouse anti-beta-Actin (Abcam, ab6276) antibodies were used to control equal loading of protein extracts. As secondary antibodies, horseradish peroxidase-conjugated anti-rabbit and anti-mouse IgGs (Santa Cruz Biotechnology, sc-2370/sc-2005) were used. PageRuler Prestained (ThermoScientific) and biotinylated Protein Ladders (Cell Signaling, 7727S) were used for protein molecular weight estimation. Detection was done using Clarity Western ECL Substrate (Bio-Rad) and the FujiFilm LAS 3000 system.

### Immunohistochemistry

Fibroblast cells were fixed with 4% paraformaldehyde in PBS. After blocking and permeabilization with 2% bovine serum albumin, 10% normal goat serum and 0.25% TritonX-100 in PBS for 60 min at room temperature, cells were incubated with primary antibodies (rabbit anti-WDR11, Abcam, ab93871, 1:300 and mouse anti-Golgin-97, Molecular Probes, A-21270, 1:50) in blocking solution for 60 min at room temperature, washed 3 times in PBS and incubated with Alexa Fluor-488 or -568 secondary antibodies (1:500; Molecular Probes). DAPI (4’,6-diamidino-2-phenylindole, 1:1000; Invitrogen) was used for nucleic acid staining. Images were taken with a Zeiss Observer Z.1 microscope equipped with an Apotome2 and HXP 120 lamp.

## Results

### Index patients show intellectual disability, microcephaly and mild short stature

Detailed clinical evaluations of all affected individuals are summarized in Table 1. Patients III-1 and III-2 from family A were 9- and 8-year-old sisters born to first cousins of Syrian descent. Both showed microcephaly, short stature and mild intellectual disability. Brain magnetic resonance imaging (MRI) in both siblings did not reveal obvious morphological abnormalities; notably there were no anomalies of pituitary gland and olfactory bulb. X-ray of the left hand revealed a slightly retarded bone age according to the Greulich-Pyle atlas in patient III-1. In both girls there were no signs of endocrinological dysfunction; in particular, hormone profiles of the hypothalamic-pituitary-adrenal axis were largely in the normal range of prepubertal girls. Echocardiography of patient III-1 was normal and there was no evidence of a congenital heart defect on clinical examination by a pediatric cardiologist in patient III-2 at the age of 5 5/12 years. In patient III-1 pronounced hypopigmentation of trunk and neck was compatible with the clinical diagnosis of vitiligo. Patient III-2 presented with strabism. Chromosomal microarray did not reveal any known disease-associated copy number variation in individual III-1, and exome sequencing was pursued in both siblings.

**Table 1:** Clinical and molecular characteristics.

	Family A		Family B	Family C		
	Patient III-1	Patient III-2	Patient II-I	Patient III-1	Patient III-4	Patient III-5
Whole-exome sequencing: *WDR11* variant (NM_018117.12)	c.1255C>T, p.Q419* homozygous	c.1255C>T, p.Q419* homozygous	c.3033_3036del, p.(D1011Efs*21) (mat); c.1439del, p.(N480Tfs*32) (pat)	c.2931+1G>A, p.(?) homozygous	c.2931+1G>A, p.(?) homozygous	c.2931+1G>A, p.(?) homozygous
Additional genetic testing	SNP array without copy number changes	n.d.	Karyotype (amnio-centesis): 46,XY, Chromosome microarray (180 K): normal	Karyotype: 46,XX	Karyotype 46, XY, no AZF microdeletion, positive *SRY* molecular testing	n.d.
Origin	Syria	Syria	Singapore	Kuwait	Kuwait	Kuwait
Age at last exam.	9 5/12 years	8 3/12 years	12 1/12 years	30 years	30 years	26 years
Height	118 cm (-2.67 SD)/9 5/12 years	104.5 cm (-2.48 SD)/6 5/12 years; 120 cm (-1,47 SD)/8 3/12 years	135.7 cm (-1.84 SD)/12 1/12 years	n.d.	160 cm (-1.98 SD)/17 11/12 years; 164 cm (-1.75 SD) /30 years	145 cm (-2.13 SD)/13 10/12 years; 166 cm (-1.47 SD)/21 years; 167.5 cm (-1.26 SD)/ 26 years
Weight	19 kg (-2.63 SD)/9 5/12 years	17.2 kg (-1.59 SD)/6 5/12 years; 20 kg (-1.80 SD)/8 3/12 years	30.9 kg (-1.61 SD)/12 1/12 years	3.1 kg (-0.62 SD)/birth	4 kg (0.89 SD)/birth, 41.5 kg (-2.9 SD)/17 11/12 years; 48.4 kg (-2.44 SD)/30 years	28.6 kg (-2.9 SD) 13 10/12 years; 45 kg (-2.84 SD)/21 years; 47.2 kg (-2.58 SD)/26 years
OFC	46 cm (-4.93 SD)/8 2/12 years	47 cm (-3.59 SD)/7 1/12 years	48 cm (-3.73 SD)/11 1/12 years	49.5 cm (-4.54 SD)/30 years	50.5 cm (-3.09 SD)/17 11/12 years; 51.5 cm (-2.43 SD)/ 30 years	47.8 cm (-4.32 SD)/13 10/12 years; 50.2 cm (-3.3 SD)/21 years; 51 cm (-2.76 SD)/26 years
Development diagnostic	Mild ID (WNV IQ 52 and KABC-II SFI 56/8 4/12 years)	Mild ID (SON-R 2^1^/_2_-7 IQ 54/6 10/12 years)	Mild ID (Wechsler Preschool and Primary Scale of Intelligence: Full Scale IQ 66 / 6 5/12 years)	Severe ID (not formally assessed); walked independently at 2 years, says repetitive syllables but has no word with meaning, follows simple commands, needs to be fed by others	Mild ID (not formally assessed); walked and talked at 1 years, completed the 9^th^ grade, has secretarial job	Mild ID (not formally assessed); walked independently at 18 months, talked suddenly at 5 years, hyperactive child, attended special classes in school, as an adult attended vocational school, can perform simple tasks, follows instructions well, enjoys cooking and helping others
Cerebral MRI	Normal (including pituitary gland and olfactory bulb)	Normal (including pituitary gland and olfactory bulb)	n.d.	n.d.	Unremarkable (30 years)	n.d.
EEG	Normal	n.d.	n.d.	n.d	Normal	Slightly slow and irregular basic activity. No focal or sharp activity.
Hormones	Normal: TSH, fT3, fT4, LH, FSH, 17-beta-estradiol, testosterone, SHBG, DHEAS, IGFBP-3, cortisol, ACTH, IGF-1	Normal: TSH, fT3, fT4, LH, FSH, 17-beta-estradiol, testosterone, DHEAS, IGFBP-3, cortisol, ACTH; SHBG slightly increased	n.d.	Regular menses	Normal: TSH, fT3, fT4, LH, 17-beta-estradiol, SHBG, DHEA-SO4, testosterone, AMH; Increased: FSH	Normal: TSH, fT3, fT4, LH, 17-beta-estradiol, SHBG, DHEA-SO4; Decreased: FSH
Other laboratory results	Normal: CBC, renal and liver function tests	Normal: CBC, renal and liver function tests	Microcytosis	n.d.	Normal: CBC, renal and liver function tests	Normal: CBC, renal and liver function tests
Other	Pronounced and extended hypopigmentation of trunk and neck, mild retardation of bone age, ophthalmologic and cardiologic examination (echocardiography and ECG) normal	Strabism and visual defect, clinical cardiologic examination normal	Bilateral exotropia, bilateral 5^th^ finger clinodactyly, brachydactyly	Anterior fontanel closed at birth, asthma (up to 4 years), kyphoscoliosis (15 years), retrognathia	Myopia and nystagmus, pink/ hyperemic optic neuropathy, high arched palate, mild microretrognathia, narrow chest, mild pectus excavatum, cubitus valgus, normal hearing	Febrile seizure at 18 months, seizure medication discontinued at 3 years, mild myopia, optic neuropathy, high arched palate, microretrognathia, narrow chest, cubitus valgus, bilateral mild sensory neural hearing loss

*CBC* complete blood count,* exam.* examination, *ID* intellectual disability, *KABC-II* Kaufman-Assessment Battery for Children-II, *n.d*. not determined, *OFC* occipital frontal circumference, *SON-R* Snijders-Oomen Nonverbal intelligence test, *WNV* Wechsler Nonverbal Scale of Ability.

Patient II-1 of family B, a 12-year-old boy, was the only child of a non-consanguineous Singaporean family of Chinese ethnicity. He showed mild intellectual disability, microcephaly and mild short stature. Ophthalmological examination revealed bilateral exotropia. Furthermore, microcytosis which was consistent with beta-thalassemia trait was present. Results from conventional karyotyping and chromosomal microarray were normal.

Patients III-1, III-4, and III-5 of family C were adult children of unaffected consanguineous parents of Kuwaiti origin and presented with intellectual disability of varying degree, microcephaly, mild short stature and variable skeletal anomalies. All were the products of unremarkable full-term pregnancies. Individual III-1, age 30 years at last examination, was the most significantly affected, having not achieved any meaningful speech, able to follow simple commands only and requiring assistance to feed herself. She had asthma as a young child and was diagnosed with kyphoscoliosis at 15 years. Her younger brothers, age 30 (III-4) and 26 (III-5) years at last examination, had more mild intellectual disabilities, both able to achieve some schooling and continue to work or volunteer in their community as adults. III-4 had surgical correction of nystagmus at 6 years and wore glasses for near-sightedness. III-5, had a single febrile seizure at 18 months and was also near-sighted. Conventional karyotyping was normal in III-1 and III-4, and III-4 had an unremarkable brain MRI at 30 years.

### WES reveals rare biallelic *WDR11* variants in all affected patients

**Fig. 1:**
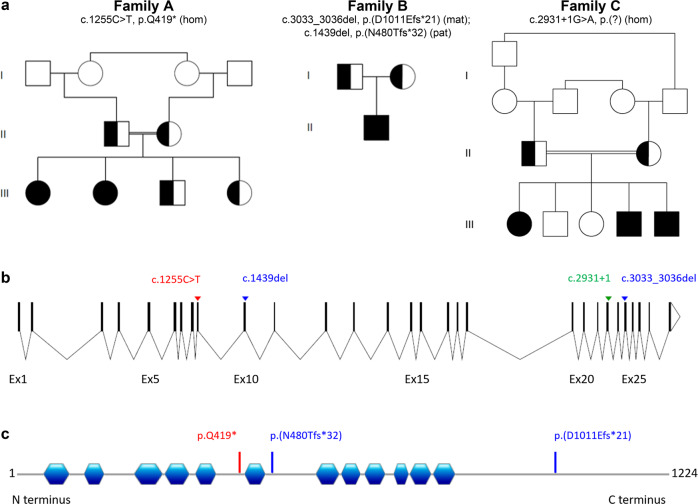
Pedigrees and schematic representation of all identified variants in this study. **a** Pedigrees of family A, B, and C. **b** Scheme of the *WDR11* gene showing the identified variants in family A (red), family B (blue) and family C (green). **c** Linear map of the WDR11 protein (NP_060587.8) indicating the twelve WDR domains (blue) as described in [1] and the identified coding variants in family A (red) and family B (blue).

All affected patients from the three families showed rare biallelic variants in the *WDR11* gene (OMIM: *606417) (Fig. 1a–c). In Family A, a homozygous nonsense-variant [NC_000010.11:g.120867130C>T, NM_018117.12:c.1255C>T p.Gln419*], which was absent from gnomAD, was identified in individuals III-1 and III-2. Sanger sequencing showed that both parents and both unaffected siblings were heterozygous carriers. In family B, a 4-bp deletion [NC_000010.11:g.120904651_120904654del, NM_018117.12:c.3033_3036del p.(Asp1011Glufs*21)] inherited from the mother and a 1-bp deletion [NC_000010.11:g.120871314del, NM_018117.12:c.1439del p.(Asn480Thrfs*32)] inherited from the father were identified. Both frameshift variants are listed with extremely low frequencies in gnomAD. The maternal variant (rs760973100) has been detected in 1 out of 251,436 alleles and the paternal variant has been detected in 4 out of 251,334 alleles in gnomAD. Based on their location both variants were subject to degradation by nonsense-mediated mRNA decay (NMDEscPredictor: https://nmdprediction.shinyapps.io/nmdescpredictor/). In family C, a homozygous splice variant [NC_000010.11:120903233G>A, NM_018117.12:c.2931+1G>A p.(?)] was detected at the canonical donor splice site of intron 23. Several in silico tools (SSF, MaxEnt, NNSplice) predicted complete disruption of the donor splice site of intron 23 and did not show any evidence for the use of a cryptic splice site. Therefore skipping of exon 23 (178 bp) is very likely which subsequently leads to a frameshift and premature stop codon. Homozygosity for the variant was identified in the three affected siblings only; both parents were heterozygous carriers and unaffected siblings were not homozygous for the variant. This rare variant is seen in gnomAD in 1 out of 251,298 alleles and is absent from the Greater Middle Eastern Variome Project.

### WDR11 protein is absent in patient fibroblasts

**Fig. 2:**
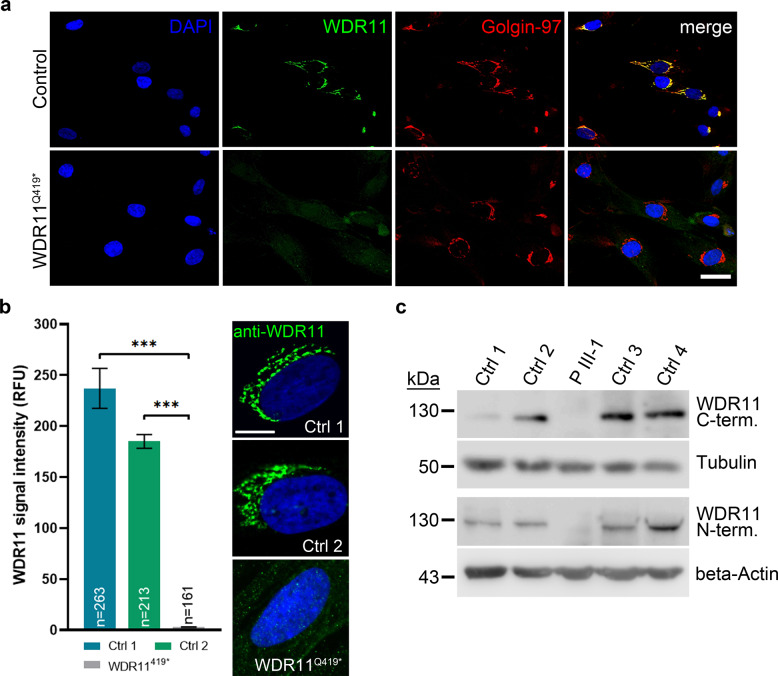
Expression analysis of the homozygous WDR11Q419* variant. **a** WDR11Q419* leads to loss of WDR11 protein in patient fibroblasts. Immunostaining of control (upper panel) and WDR11^Q419*^ fibroblasts (lower panel) with antibodies against WDR11 (green; Abcam, ab93871) and the Golgi/TGN marker Golgin97 (red; Molecular Probes, A-21270). Control cells show strong juxtanuclear WDR11 staining, while WDR11^Q419*^ fibroblasts only show weak and unspecific background labeling. Scale bar, 10 µm. **b** Quantification of WDR11 signal intensities. Small images to the right show exemplary cells of two different control (Ctrl 1, Ctrl 2) and WDR11^Q419*^ fibroblasts stained with anti-WDR11 antibodies (ab93871, green) used for quantification. Nuclear counterstain: DAPI. Data represent mean ± SEM, *n* number of analyzed cells. ***P < 0.0001; One-way ANOVA, Bonferroni’s multiple comparisons test. Scale bar, 20 µm. RFU: relative fluorescence units. **c** Western blot analyses show WDR11 expression in 4 independent control fibroblasts (130 kDa) but complete absence in patient III-1 of family A (2 independent experiments). No truncated WDR11 protein fragments were evident in patient’s lysate (see also Supplementary Fig. 1). *N-term* N-Terminus, antibody recognizing epitope aa268–348, *C-term* C-terminus, antibody recognizing epitope aa1174–1224.

Immunofluorescence staining of cultured fibroblasts showed strong juxtanuclear WDR11 staining in control fibroblasts, while WDR11^Q419*^ fibroblasts only showed a cell-ubiquitous background labeling (Fig. 2a). Co-staining with Golgin-97, a trans-Golgi network (TGN) marker, showed localization of wildtype WDR11 to the TGN. In contrast, WDR11^Q419*^ fibroblasts lose this characteristic colocalization pattern, indicating WDR11 protein loss. Quantification of WDR11 signal intensities in 100–200 cells per fibroblast line (ImageJ/Fiji software, version 1.53c; http://imagej.nih.gov/ij) highlights the drop in WDR11 signal in WDR11^Q419*^ fibroblasts from patient III-1 of family A (Fig. 2b).

To prove loss of WDR11 protein, Western blot analysis was performed in whole-cell lysates of cultured fibroblasts of individual III-1 of family A using antibodies against N- and C-terminal epitopes of WDR11. Complete absence of WDR11 protein without indication of a truncated variant protein expected to be 47 kDa in size (see Supplementary Fig. 1 for original, uncropped blots) was demonstrated for patient III-1, whereas controls showed a band at 130 kDa, as expected for full-length WDR11 (Fig. 2c).

## Discussion

In this study, we report for the first time three unrelated families with the core features of intellectual disability, microcephaly, and mild short stature in whom genetic analysis revealed biallelic loss-of-function variants in *WDR11* (Table 1).

Collaboration of our three research groups investigating three different families with biallelic loss-of-function variants in *WDR11* and a similar phenotype had been made possible through Genematcher [5]. *WDR11* is a member of the WD repeat containing family and variants of WDR proteins have been associated with various human diseases including neurological disorders, ciliopathies, endocrine disorders and cancer [7].

*WDR11* was first suggested as candidate gene for CHH by the definition of the chromosomal breakpoint of a balanced t(10;12) translocation in a patient with Kallmann syndrome. The breakpoint in chromosome 10 was located 547 kb from the 5’ end of *WDR11* [1]. In this patient, a 20% reduction of the mRNA and 10% of WDR11 protein analyzed by RT-qPCR and western blot, respectively, was suggested as a potential position effect that impairs WDR11 expression. Targeted sequencing of the gene in further CHH/Kallmann patients revealed five rare heterozygous missense variants in *WDR11* in six patients in the same study. However, parental segregation analysis of the identified variants was not presented. Furthermore, a patient with combined pituitary hormone deficiency was found to have a maternally inherited splice-site variant in *WDR11* (NC_000010.11:g.120860107A>G; NM_018117.12:c.353-2A>G), that presumably leads to a 58 amino acid deletion/1 amino acid insertion [8]. Another maternally inherited *WDR11* missense variant with reduced penetrance has been associated with pituitary dysgenesis, growth hormone deficiency and obesity in two brothers [3]. The data at least argue for highly variable clinical penetrance and/or expressivity of heterozygous *WDR11* variants associated with CHH/Kallmann syndrome.

In the parents of our patients – heterozygous carriers of *WDR11* loss-of-function alleles – there were no obvious signs of CHH, although detailed endocrinological examination had not been performed. Many descendants in two of three families further argue against reduced fertility in carriers of heterozygous *WDR11* loss-of-function variants. The finding that we did not observe obvious clinical signs of CHH in any of the heterozygous *WDR11* carriers questions an association between heterozygous *WDR11* variants and CHH [1, 3, 8], however, specific variants in *WDR11* might exert different effects in humans.

*WDR11* is located within the region that is associated with 10q26 microdeletion syndrome which is associated with neurodevelopmental impairment, microcephaly, facial dysmorphism, growth retardation, cardiac defects, coloboma, and urogenital abnormalities [9–11]. The lack of clinical signs in heterozygous carriers of *WDR11* loss-of-function variants in our families argues against *WDR11* being a candidate gene of 10q26 microdeletion syndrome.

In fibroblasts of individual III-1 of family A, we demonstrated a complete loss of WDR11 protein. Bi-allelic loss-of-function of WDR11 has already been intensively examined in mice, zebrafish and the human embryonic olfactory GnRH neuroblast cell line FNCB4-hTERT [3]. *Wdr11*-knockout mice exhibited mid-gestation embryonal lethality, growth retardation and significant developmental defects like shortened limbs and hypoplastic skeleton. Moreover, *Wdr11*-null mice showed features of holoprosencephaly (HPE) that included microcephaly, hypotelorism, microphthalmia or anophthalmia, further craniofacial midline defects or signs of lobar HPE. Other abnormalities of *Wdr11*-knockout mice were cardiac defects, dysmorphogenesis of the pituitary gland, hypoplasia of the reproductive organs and infertility. In zebrafish, endogenous *wdr11* knockdown led to microcephaly and aberrant head cartilage formation, microphthalmia, curved body axis, motility defects, narrow trunk and melanocyte disorganization. The phenotype associated with *WDR11* loss-of-function variants in humans shows overlap to that found in *Wdr11*-knockout mice and fish with regard to microcephaly and growth retardation. Furthermore, similar to the phenotype observed in *Wdr11*-knockout mice, individuals with *WDR11* biallelic loss-of-function variants showed i.a. anomalies of the visual (strabism, visual defects, nystagmus, optic neuropathy) and skeletal (brachydactyly, fifth finger clinodactyly, kyphoscoliosis, narrow chest, high arched palate and (micro-)retrognathia) system. In contrast, there was no evidence of a congenital heart defect or brain malformation in our patients. The adult affected brothers of family C showed largely normal profiles of sexual hormones and the affected sister showed regular menses and therefore no signs of CHH. In childhood, diagnosis of CHH is difficult, but undetectable levels of FSH might indicate CHH [2]. In both siblings of family A, comprehensive analyses of hormones and MRI of hypophysis were normal and therefore exclude at least combined pituitary hormone deficiency or pituitary dysgenesis. In homozygous *Wdr11*-knockout mice the associated spectrum was broad and the associated phenotype was variably expressed. Only 31% of the mouse embryos showed a heart defect and 33% of the pups showed hydrocephalus. Skeletal defects were found in 32% of *Wdr11*-knockout mice and eye defects in 15% while infertility was found in 75% of pups [3]. Due to the currently small number of patients with biallelic loss-of-function variants in *WDR11* the full clinical spectrum and the frequency of associated anomalies in humans need to be elucidated in further studies. However, our preliminary data suggest that the associated phenotype in humans seems to be considerably milder than in *Wdr11*-knockout mice. Early lethality or a more severe phenotype in mice than in humans is in accordance with several other mouse mutants of different human ciliopathy genes [12].

Besides of its role in Hh signaling and ciliogenesis, *WDR11* has been implicated in endosome-to-trans-Golgi network (TGN) vesicular trafficking [13], i.e., AP1 dependent cargo proteins are no longer guided to the TGN but accumulate on the plasma membrane in the absence of WDR11. Human fibroblasts showed strong juxtanuclear WDR11 staining at the TGN (Fig. 2a), while fibroblasts from individual III-1 of family A did only show weak and unspecific background labeling. The location of WDR11 in humans at the TGN suggests that WDR11 may also play a role in endosome-to-TGN vesicular trafficking and absence of WDR11 staining in patient III-1 of family A further confirms loss of WDR11 in our patient.

In conclusion, our data suggest that biallelic loss-of-function variants of human *WDR11* are associated with a distinct phenotype that includes pronounced microcephaly, mild short stature and intellectual disability of variable degree.

## Supplementary information

Supplement to Figure 2

## Data Availability

The datasets generated and/or analyzed during the current study are available from the corresponding author on reasonable request.
