# Supplementary material for: Biallelic loss-of-function variants in WDR11 are associated with microcephaly and intellectual disability
Source: Eur J Hum Genet. 2021 Aug 20;29(11):1663–8. doi: 10.1038/s41431-021-00943-5 (PMC8560748; doi:10.1038/s41431-021-00943-5)
Supplement: Supplementary file 1 — Supplement to Figure 2 [file 41431_2021_943_MOESM1_ESM.pdf]

Supplementary information

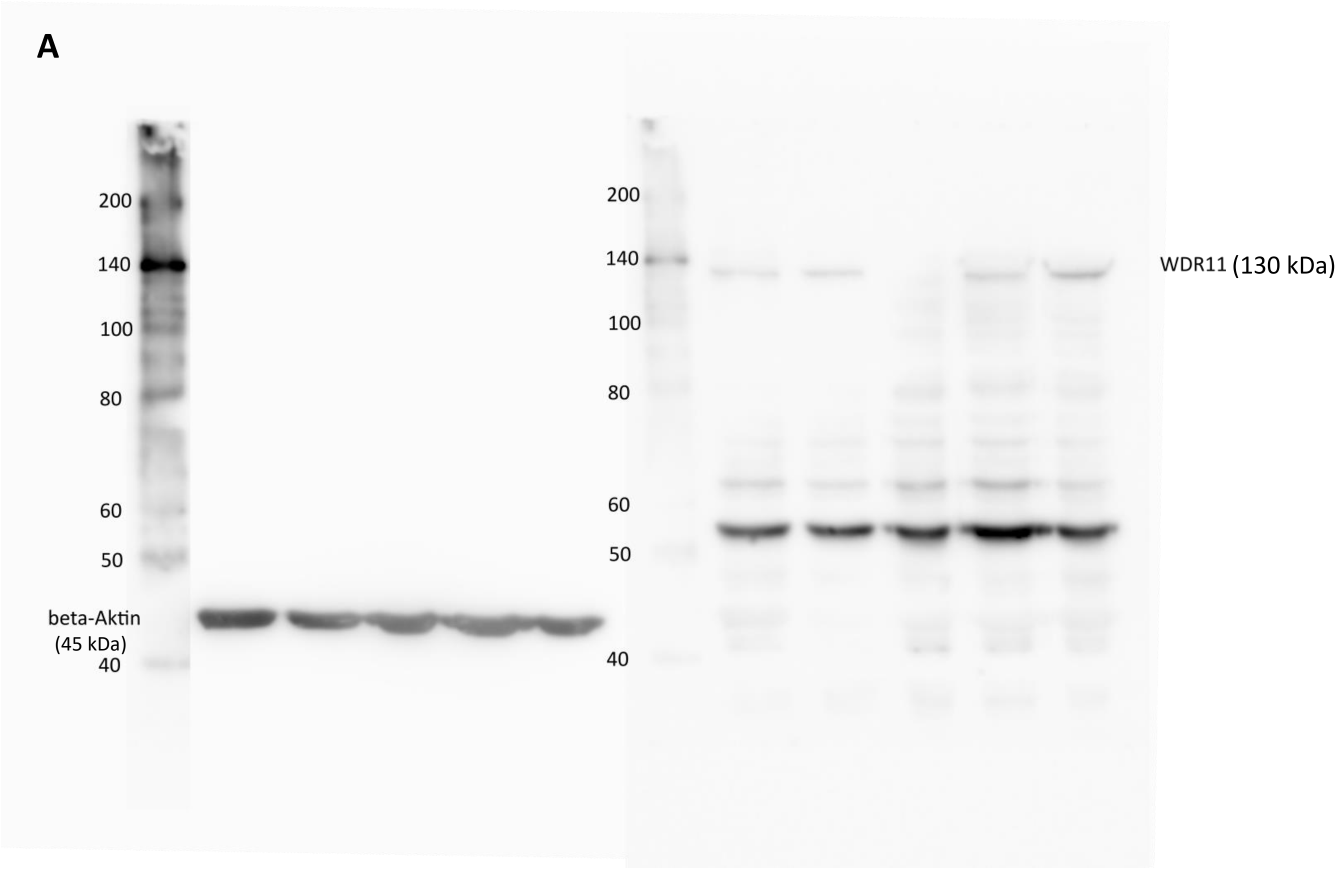

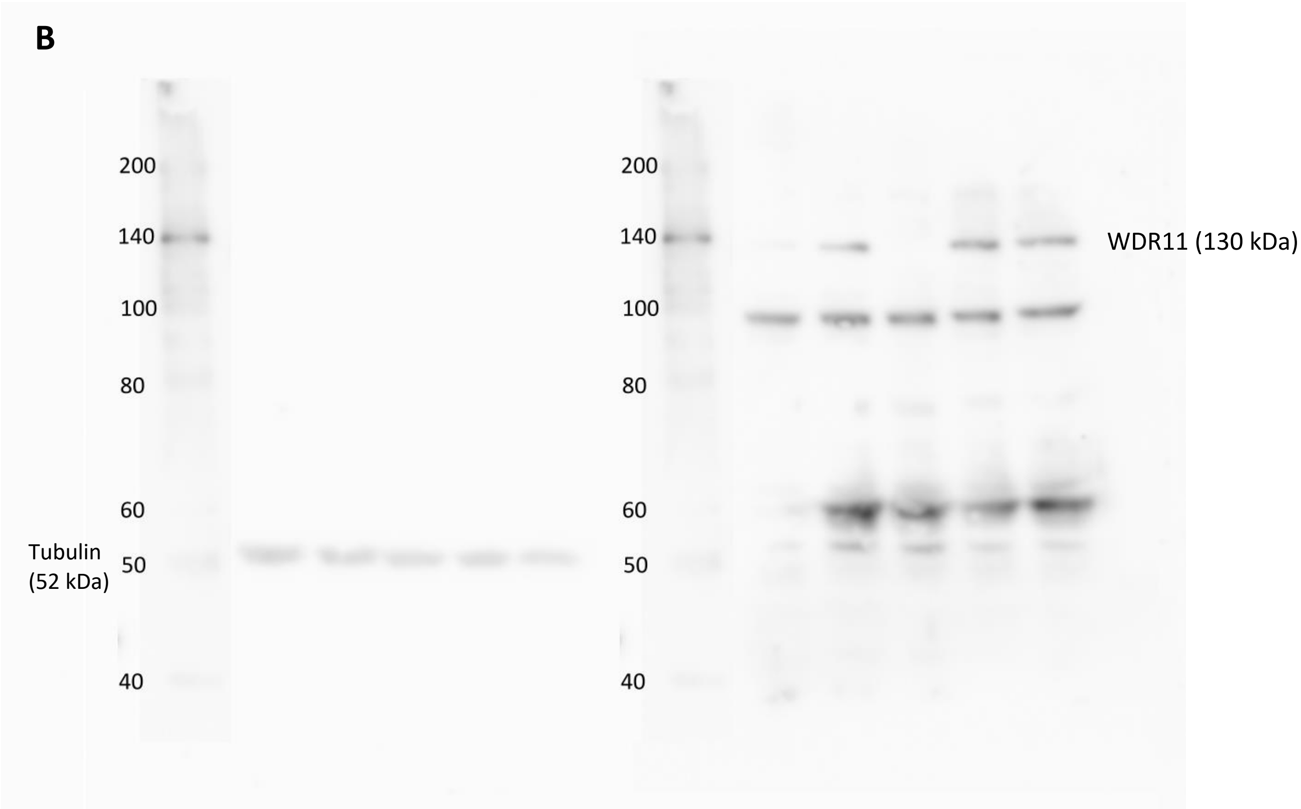

Supplement to Figure 2: Full, uncropped blots from Figure 2c. **A**) anti-WDR11 staining using the N-terminal antibody (right blot) with the corresponding loading control (left blot, anti-beta-Actin). **B**) anti-WDR11 staining using the C-terminal antibody (right blot) with the corresponding loading control (left blot, anti-Tubulin). Sample loading from left to right: Ctrl 1, Ctrl 2, Patient III-1, Ctrl 3, Ctrl 4.
